# Unravelling the long-term river health status of Kruger National Park Rivers using macroinvertebrate-based monitoring

**DOI:** 10.1007/s10661-025-14343-5

**Published:** 2025-07-14

**Authors:** Hendrik Sithole, Samuel N. Motitsoe, Thendo Mutshekwa, Musa C. Mlambo

**Affiliations:** 1 Conservation Services Division, Scientific Services, South African National Parks (SANParks), Kimberley, South Africa; 2 School of Animal, Plant and Environmental Sciences, University of the Witwatersrand, Private Bag 3, Johannesburg, 2050 South Africa; 3 Albany Museum, Department of Freshwater Invertebrates, Somerset Street, Makhanda (Grahamstown), 6139 South Africa; 4 Institute for Water Research, Rhodes University, Makhanda (Grahamstown), 6139 South Africa; 5 Department of Zoology and Entomology, Rhodes University, Makhanda (Grahamstown), 6140 South Africa; 6 South African Institute for Aquatic Biodiversity, Makhanda, 6140 South Africa

**Keywords:** River biomonitoring; South African Scoring System version 5 (SASS5), Average score per taxon (ASPT), Present ecological status (PES), Anthropogenic activities

## Abstract

The burgeoning population growth and landscape developments over the last decades have had a profound effect on natural resources including riverine water quality and quantity. This resulted in increased pollution events, loss of aquatic biodiversity and the introduction of alien invasive species, which cumulatively have transformed freshwater ecosystem structure, processes and functions. Macroinvertebrate-based biomonitoring, like the South African Scoring System version 5 (SASS5), is a fast and effective way to assess water and habitat quality. However, studies reporting long-term biomonitoring are rare in many Global South systems. Also, river health within protected areas is generally regarded better than those outside, with the park potentially offering refugium and ideal conditions for rivers to recover. However, Kruger National Park (KNP) rivers get impacted by mining and domestic sewage upstream, before entering the park, and it is not clear how much remediation happens as the rivers flows downstream within the park. The current study was initiated to investigate the long-term river health status of four major river systems flowing across the iconic KNP, using SASS5 biomonitoring and selected physico-chemical parameters. Our results showed that SASS5 indices showed difference in their sensitive to anthropogenic impacts, both in space and time. Long-term dataset revealed that the ability of the rivers to recover from poor to good quality (and vice versa) was quite rapid, and also some rivers were progressively declining (e.g. Olifants and Crocodile rivers) while others were improving (e.g. Luvuvhu and Sabie rivers). Consequently, SASS5 appeared to be a useful tool in assessing the long-term health of KNP rivers, providing valuable insights into the overall ecological conditions and water quality of these important aquatic ecosystems that will assist in the park water resource management strategies. Further, our results demonstrated high sensitivity and quick response of macroinvertebrates to disturbance, and an improvement potential as the rivers transversed the park.

**Supplementary Information:**

The online version contains supplementary material available at 10.1007/s10661-025-14343-5.

## Introduction

Aquatic environments are the most threatened ecosystems globally (Darwall et al., 2011; Ficke et al., 2007; Malmqvist & Rundle, 2002). Though protected areas are good at protecting terrestrial biodiversity and some aquatic populations (Acreman et al., 2020), it appears that rivers in protected areas, like national parks, are not immune from anthropogenic influences (Hermoso et al., 2016; Riddell et al., 2019, 2022; Roux et al., 2002). Generally, the record of protected areas conserving aquatic biodiversity and ecosystems is mixed (Valentim et al., 2024), as they were largely created to support terrestrial megafauna, with aquatic biodiversity coming as an afterthought. Consequently, some aquatic species and populations are benefitting while others are not (Nogueira et al., 2021; Zamora-Marín et al., 2016). The longitudinal and lateral connections of rivers (Allan, 2004) means that any pollution event happening upstream of the park will potentially have profound effects on the downstream (Shikwambana et al., 2021). This poses a real problem for the conservation of riverine biodiversity, especially in protected areas (Riddell et al., 2019; Roux et al., 2002). Further, anthropogenic activities such as water abstraction and damning happening upstream affect the extent and frequency of drought-flood dynamics downstream (Malherbe et al., 2020; Rountree et al., 2000).

Long-term studies and historical records provide a unique opportunity for ecologists to investigate the pressing temporal changes on biodiversity due to climate and anthropogenic impacts. For example, Haase et al. (2023) using a continent-wide long-term dataset from Europe revealed interesting patterns of aquatic invertebrate recovery and loss over decades. However, such studies and programmes are generally lacking when it comes to aquatic ecosystems in the Global South (Bickerton, 1995; Chessman, 2012; Resh et al., 2013). This could be attributed to the lack of funding to support long-term studies, scarcity of taxonomic expertise and general short-term life cycle of most research projects or funding, which is largely linked to student dissertations (Gerecke et al., 2011; Resh et al., 2004). Changes that happen over longer periods, like alternating drought, floods events and landscapes developments are challenging to assess with the conventional short-term studies, thus requiring long-term studies (i.e. consistent observation of at least over a decade) (Weeks et al., 1996). Long-term studies not only tell us about the biology and ecology of the target organisms but can also demonstrate response potential, duration and the extent of recovery after disturbance (Resh et al., 2013).

Biological monitoring (hereafter biomonitoring) offers a comprehensive image of the water quality and wellbeing of lotic ecosystems (Cairns & Pratt, 1993; Carter et al., 2017). Benthic macroinvertebrate assemblage, spending some stages of their lives in the aquatic environment, with relative limited mobility (compared to fish and birds) are an ideal candidate for biomonitoring, as they effectively integrate and reflect environmental disturbances on the ecosystem (Buss et al., 2015; Feio et al., 2021; Ollis et al., 2006). Consequently, macroinvertebrate-based biomonitoring approaches are quite common and are used throughout the world (Buss et al., 2015; Feio et al., 2021; Sumudumali & Jayawardana, 2021). In South Africa, the South African Scoring System version 5 (SASS5), is a macroinvertebrate-based biomonitoring tool used to assess river health and water quality (Dallas, 2021; Dickens & Graham, 2002). SASS5 evaluates the sensitivity of aquatic invertebrate families to pollution, assigning scores from 1 (most tolerant) to 15 (most sensitive) (Dickens & Graham, 2002; Odume & Mgaba, 2016). Ultimately, two critical indices are produced: the SASS score and Average Score Per Taxon (ASPT). The SASS score is the sum of the sensitivity/quality values for all families present in the sample, whereas on the other hand, ASPT is determined by dividing the SASS score by the number of present/observed taxa. These measures are complimentary, as they have differential biases and sensitivities (Dickens & Graham, 2002; Ollis et al., 2006).

The South African Kruger National Park (KNP) is one of the largest game reserves in Africa (SANParks, 2018). For its water resources, it is dependent upon its four major rivers that run across the park into Mozambique, i.e. the Luvuvhu, Olifants, Sabie and Crocodile rivers. All these rivers are subjected to various landscape developments arising from upstream tributaries and having a significant impact to the KNP river health and water quality (Riddell et al., 2019, 2022; Shikwambana et al., 2021). The KNP rivers are prone to alternating drought and floods due to their geographical location and interactions with human activities (Malherbe et al., 2020; Mazibuko et al., 2021; Odiyo et al., 2015; Pollard et al., 2011; Weeks et al., 1996). Long-term studies are useful in documenting the temporal changes through biological indicators particularly for such important systems in Southern Africa with such unique landscape and environmental stressors. Although aquatic macroinvertebrates have been studied in the park (Majdi et al., 2022; Weeks et al., 1996), there are virtually no long-term biomonitoring studies of aquatic macroinvertebrates in KNP (Marr et al., 2017a, 2017b). As such, it is critical to undertake a long-term biomonitoring study in KNP rivers to track and map the spatio-temporal anthropogenic impacts in the main rivers of the park.

This study aimed to assess the spatio-temporal variation in river health and water quality of the KNP rivers using the macroinvertebrates-based biomonitoring SASS5 protocol. We conducted annual biotic assessments of these four major rivers for a period of 10 years (2010–2019), recording both SASS score and ASPT, including rapid habitat assessment and water quality variables as mandated (Odume & Mgaba, 2016). To this end, we expected that the macroinvertebrates-based biomonitoring technique will be sensitive to activities like drought or flooding events happening during the study, as well as provide reliable biomonitoring status of the KNP river health and water quality changes over time and space.

## Materials and methods

### Study area

**Fig. 1:**
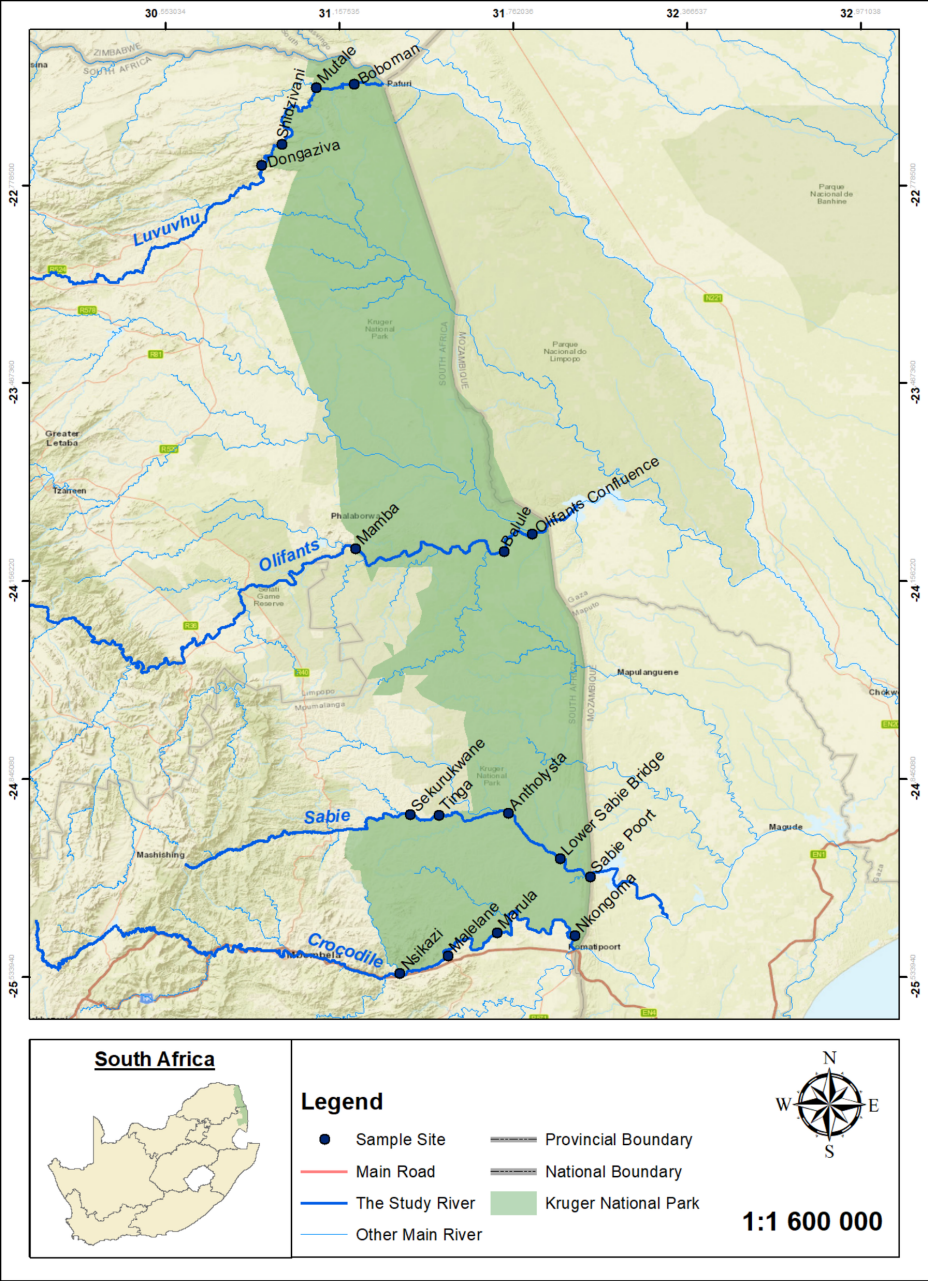
The Kruger National Park boundaries, and the four rivers and their sites sampled during 2010–2019, South Africa

This study was conducted from 2010 to 2019 on four rivers, namely Crocodile, Sabie, Olifants and Luvuvhu rivers within KNP boundaries (Fig. 1). All the rivers flow into the KNP from the western boundary and exit at the eastern border into Mozambique (Pollard et al., 2011). The park experiences summer rainfall between October and March (Pollard & du Toit, 2011), with rainfall and discharge peaking in January and February (DWAF, 2004a). However, Knight and Evans (2022) reported that average rainfall patterns rapidly decline within the park resulting to reduced discharge from tributaries originating within the park thus compromising the dilution effect from upstream counterparts.

The Crocodile River is the southernmost river in the KNP and stretches 115 km within the park before merging with the Nkomati River, also located within the park (Fig. 1). The river discharges about 364 million m^3^ of water per annum (DWAF, 2004a) into the Nkomati catchment system (Stone-Jovicich et al., 2011), with about 36% of the Nkomati River length found within the KNP (Deksissa et al., 2004). The 70-km long Nsikazi River is the only perennial tributary to the Crocodile River which also joins within the park. Four sites were selected and sampled in the Crocodile River and thus included (from the western to eastern boundary) the Malelane (− 24.46079; 31.53466), Nsikazi (− 25.5222; 31.36848), Marula (− 25.37988; 31.70584) and Nkongoma (− 25.391201; 31.97626) (Fig. 1). The sites consisted of riverbeds dominated by cobbles (Malelane and Marula), sand (Nsikazi) and bedrock (Nkongoma) substrates with sparse riparian and aquatic vegetation observed. This river is impacted by untreated wastewater effluent from municipal and agricultural sources (Malakane & Maphanga, 2024), heavy metal pollutants (van Gessellen, 2015), and the presence of invasive species such as *Cherax quadricarinatus* (Hoffman et al., 2017).

The Sabie River is the second southern KNP perennial river with a length of about 110 km in the park (Pollard et al., 2011). It discharges about 293 million m^3^ of water per annum (DWAF, 2004a) also into Nkomati River catchment (Knight & Evans, 2022) (Fig. 1). The Sand River is the only perennial tributary to the Sabie River in the park. Five sites we sampled in the Sabie River including the Sekurukwane (− 24.97000; 31.406390), Tinga (− 24.97067; 31.50496), Antholysta (− 24.96495; 31.74455), Lower-Sabie bridge (− 25.12141; 31.92519) and the Sabiepoort (− 25.18470; 32.03080) (Fig. 1). All sites consisted of riverbeds dominated by bedrock with sparse riparian and aquatic vegetation. The Sabie River is primarily affected by sediment deposition and changes in geomorphology due to historical floods and droughts (Rountree et al., 2000).

The Olifants River runs through the middle of the park, serving as a provincial boundary between Mpumalanga and Limpopo Provinces, spanning approximately 100 km (Fig. 1). The Olifants River discharges approximately 699 million m^3^ annually within the catchment (De Villiers & Mkwelo, 2009) before flowing into Mozambique. Approximately 40% of the Olifants River’s length flows through the park (Carvalho et al., 2009), with Klasirie, Timbavati and Letaba rivers as the main perennial tributaries originating outside KNP borders but joining the main stem within the park. Three sites were selected and sampled within the Olifants River, i.e. Mamba (− 24.04244; 31.21503), Balule (− 24.0529; 31.72998) and the Olifants confluence (− 23.99173; 31.82709) (Fig. 1). All three sites were mostly dominated by bedrock with patches of cobbles and mudflats substrates. This river is severely impacted by heavy metal pollution from upstream mining activities (van der Merwe, 1992; van Gessellen, 2015).

Lastly, the Luvuvhu River is the northernmost perennial river in the KNP and stretches approximately 84 km within the park. It joins the transnational Limpopo River inside the park close to the Mozambique and Zimbabwe international borders (Fig. 1). Luvuvhu River contributes about 156 million m^3^ discharge per annum into the Limpopo catchment (Odiyo et al., 2015) and with the Mutale River originating outside the park been the only perennial tributary of the Luvuvhu River, with about 42% of its total length running in the park (Kleynhans, 1996). Four sites were sampled including the Dongaziva (− 22.70976; 30.88843), Shidzivani (− 22.63492; 30.95915), Mutale (− 22.42773; 31.07745) and Boboman (− 22.42773; 31.20942) (Fig. 1). All the sites consist of riverbeds dominated by bedrock (Dongaziva), bedrock and cobles (Shidzivani and Mutale) and sand (Boboman). The Luvuvhu River has been impacted by riparian habitat degradation (Kleynhans, 1996), altered hydrological connectivity and persistent droughts (Griscom, 2007).

### Data collection

#### Environmental variables

At each site and sampling event, normally early September (spring season), pH and electrical conductivity (EC; µS/m) measurements were collected using a portable EC500 pH and conductivity metre from Extech Instruments®. Additionally, river flow data (hereafter, flow m^3^/sec) for each river system were taken from the KNP weir stations, this included daily river flow data a month before sampling took place (from October to September of 2010–2019), to accommodate the flow conditions before sampling.

After SASS5 assessment, habitat composition at each site was assessed following the procedure described by Dallas (2005). At each site, the abundance of each biotope was assessed and scored: 0 when such biotope was absent, 1 when it was rare, 2 sparse, 3 common, 4 when it was abundant and 5 when the entire site was mainly composed of a biotope concerned (Dallas 2005).

#### Biological data

To determine the river health and water quality status for the four KNP rivers, the SASS5 macroinvertebrate-based biomonitoring protocol was conducted. Briefly, macroinvertebrates samples were collected using the kick sampling method described by Dickens and Graham (2002). Each habitat biotope, i.e. stones in and out of current (stones), gravel-sand-mud (GSM) and aquatic and marginal vegetation (hereafter vegetation) were vigorously sampled separately following the prescribed time intervals of 3 min for kicking and overturning the stones in-and-out of current with an addition 1 min if the was a bedrock on-site, 1 min for the GSM biotope and two square meters area for sweeping floating, submerged, emergent and over-hanging aquatic and marginal vegetation (Dickens & Graham, 2002). Thereafter, macroinvertebrates from each biotope were identified and counted to family level within 15 min intervals using aquatic invertebrates of South African rivers field guides by Gerber and Gabriel (2002). All sampling and macroinvertebrates sorting and identification were performed on-site by an accredited SASS5 practitioner.

### Statistical analysis

Two-way analysis of variance (2-way ANOVA) with Tukey post hoc HSD test was used to assess any significant differences in environmnetal variables with rivers (*n* = 4) and sampling years (*n* = 10) as factors, after testing the data for homogeneity of variances (Levene’s test, *P* < 0.05) and normality (Shapiro–Wilk test, *P* > 0.05). To investigate SASS score and ASPT indices response to rivers and years, a Linear Mixed-Effects Model using the ‘lme4’ package (lmer function) and post hoc Tukey test package ‘multcomp’ (glht function, Bonferroni adjusted) was employed. Sites were treated as a random effect, whereas SASS indices and River + Years were fixed effects. Model assumptions such as normality and homogeneity were validated by inspecting residual diagnostic plots (e.g. KS and Levene tests) found in the DHARMa package (Hartig, 2022). The SASS Scores and ASPT from the present study were further used to determine the Present Ecological Classification status which was developed by Kleynhans et al. (2005). The Present Ecological Status (PES) provides a standardized measure of river health by comparing current biological conditions to reference conditions that represent minimal human impact. In this study, PES was determined using SASS5 metrics (SASS score and ASPT), which are widely applied biomonitoring indices based on macroinvertebrate community composition. These indices are sensitive to changes in water quality and habitat integrity, making them reliable indicators of ecological health.

Site-specific reference conditions were established by Todd and Thirion (unpublished reports, 2011 and 2013), following the guidelines from Thirion (2007), and take into account both regional (ecoregional) and longitudinal (upstream–downstream) differences. By comparing current SASS5 metrics to these reference scores, we calculated PES as a percentage similarity. For example, a similarity score of 90–100% indicates little to no ecological degradation and is classified as Class A. Class B represents 80–89% similarity, Class C: 60–79%, Class D: 40–59%, Class E: 20–39% and Class F: < 39% similarity (Kleynhans et al., 2005).

Multiple linear regression analysis was used to examine which environmental variables (predictor variables) influenced SASS5 metrics (explanatory variables). The model included all four measured variables including pH, EC, flow and habitat. The StepAIC function from the package MASS (Venables & Ripley, 2002) was employed to perform forward–backward selection of the predictor variables and the best model, with all four variables showed the lowest Akaike’s information criterion (AIC) score and were selected. All statistical analyses were performed using R v4.4.1 (R Core Team, 2024).

## Results

###  Environmental variables

A two-way ANOVA results revealed that pH (River: *F* = 24.93, df = 3, *P* < 0.0001; Year *F* = 8.05, df = 8, *P* < 0.0001); EC (River: *F* = 200.59, df = 3, *P* < 0.001; Year *F* = 3.03, df = 8, *P* < 0.01) and flow (River: *F* = 47.96, df = 3, *P* < 0.0001; Year *F* = 21.54, df = 8, *P* < 0.0001) were significantly different between KNP rivers and sampling years, with only habitat composition (River: *F* = 1.36, df = 3, *P* > 0.05; Year *F* = 4.79, df = 8, *P* < 0.0001) showing no significant differences between rivers, though different temporally (Table 1).

**Table 1:** A two-way analysis of variance results for selected environmental variables collected from four major rivers systems in the Kruger National Park Rivers during the 2010–2019 study period

Variables	Factors	DF	Mean squares	*F*-value	*P*-value
EC	River	3	1,614,269	200.59	*P* < 0.0001
	Year	8	24,365	3.03	*P* < 0.01
pH	River	3	3.133	24.93	*P* < 0.0001
	Year	8	1.011	8.046	*P* < 0.0001
Flow	River	3	99.83	47.96	*P* < 0.0001
	Year	8	44.84	21.54	*P* < 0.0001
Habitat	River	3	131.3	1.36	*P* > 0.05
	Year	8	463.1	4.79	*P* < 0.0001

Significant differences in pH were observed in the Crocodile River in 2014 and 2019 (Figure S1A), in the Luvuvhu River between 2014, 2016 and 2019 (Figure S2A), in the Olifants River in 2012 (Figure S3A), and in the Sabie River in 2018 and 2019 (Figure S4A). Overall, pH levels were highest in the Olifants, followed by the Crocodile, while the Sabie and Luvuvhu rivers recorded the lowest values, similar trends were noted for EC. EC was significantly higher in the Crocodile River in 2014, 2016 and 2019 (Figure S1B), in the Luvuvhu River in 2015 (Figure S2B), lower in the Olifants River in 2010 (Figure S3B), and variable in the Sabie River during 2011, 2013 and 2015 (Figure S4B).

All four rivers showed declining flow over the 10-year period, with the Olifants River having the highest and the Luvuvhu River the lowest mean flow (Figures S1–S4C). Flow was significantly higher in the Crocodile (2010, 2011, 2013); Luvuvhu (2010, 2014, 2016); Olifants (2010, 2011) and Sabie rivers (2011, 2013, 2014). Regarding habitat composition, the Sabie River exhibited the most diverse biotopes, while the Crocodile River had the lowest. Significant changes in habitat were observed in the Crocodile River in 2014, 2016, 2018 and 2019 (Figure S1D). No significant changes were observed in the Luvuvhu and Olifants rivers (Figures S2D, S3D), while the Sabie River showed shifts post-2013, particularly in 2014 and 2015–2017 (Figure S4D).

### SASS scores, ASPT and present ecological status of KNP rivers

**Fig. 2:**
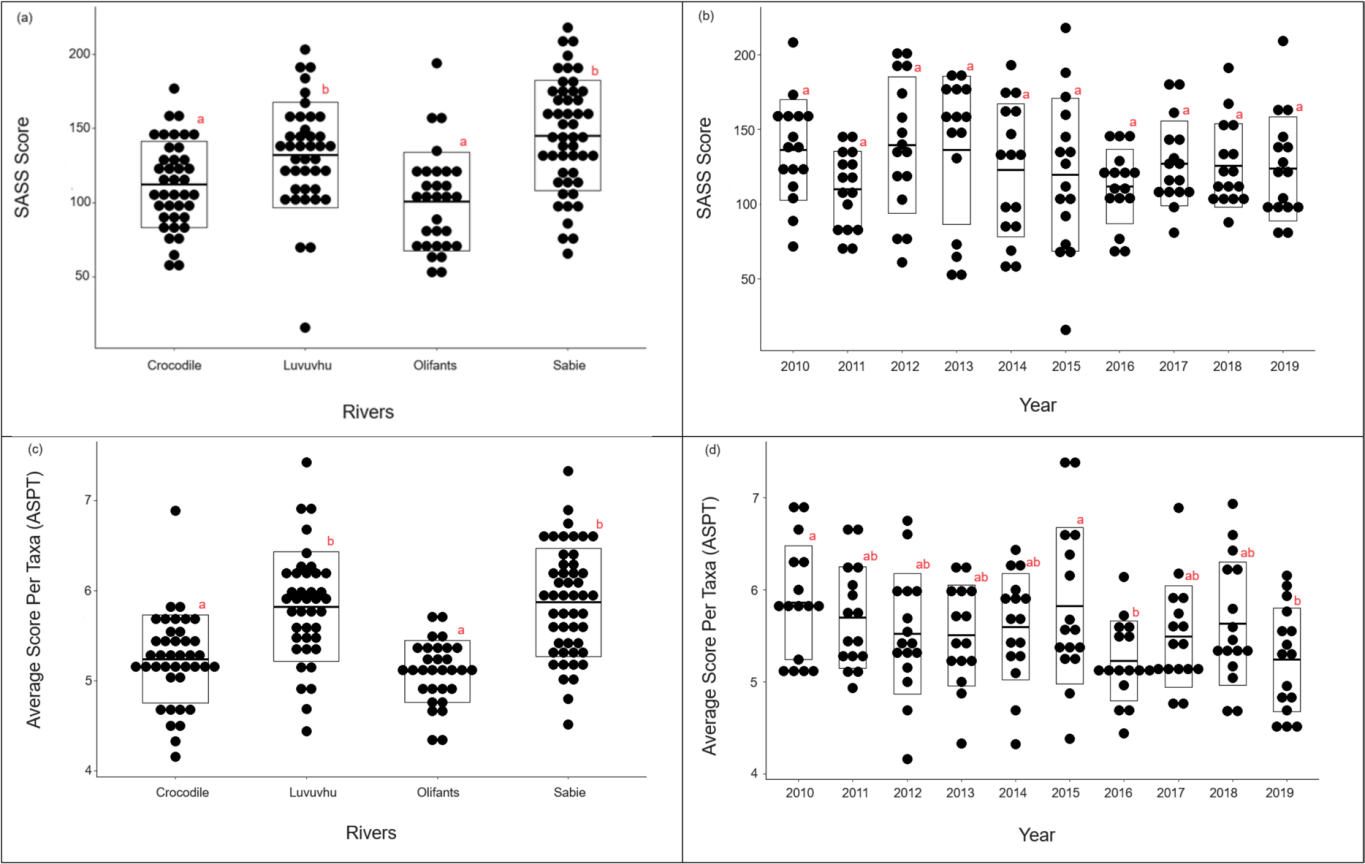
South African Scoring System indices SASS score by river (**a**), SASS score by year (**b**), ASPT by river (**c**), and ASPT by year (**d**), between the four main rivers and sampling period (2010–2019). The solid line represents the mean and the upper and lower box line represent standard deviation. Each letter represents significance difference

Spatially, SASS scores were significantly different between rivers, were multiple comparisons test with Bonferroni showed SASS scores between Luvuvhu and Crocodile rivers (*Z* = 2.76, *P* < 0.05); Sabie and Crocodile rivers (*Z* = 4.22, *P* < 0.001) and Olifants and Luvuvhu rivers (*Z* = 6.05, *P* < 0.001) to be significant (Table 2; Fig. 2A). Olifants River showed the lowest SASS score in comparison to the rest of the rivers (Fig. 2A). On average, there was a temporal decrease in SASS scores, but this trend was not significant (*Z* = − 0.93; *P* = 0.36) (Table 2; Fig. 2B). In contrast to SASS scores, ASPT was different both in space and time (Table 2; Fig. 2A and B). Luvuvhu and Crocodile (*Z* = 5.09; *P* < 0.0001); Sabie and Crocodile (*Z* = 5.98; *P* < 0.0001); Olifants and Luvuvhu rivers (*Z* = − 6.02; *P* < 0.0001) were significantly different from each other (Table 2; Fig. 2C). Similar to the SASS score, there were temporal changes in ASPT observed, with an overall decrease over time and this trend was significantly different (*Z* = − 295; *P* < 0.01) (Table 2). The Crocodile and Olifants together with Sabie and Luvuvhu rivers overall showed no difference in SASS scores and ASPT respectively (Fig. 2A and C).

**Table 2:** Linear Mixed-Effects Models results showing the effect of rivers and the sampling period (years) on SASS score and ASPT indices from four main rivers systems of the Kruger National Park, South Africa

Predictors	N	SASS			ASTP		
		Estimate (± SE)	*Z*	*P*	Estimate (SE)	*Z*	*P*
**Rivers**							
Crocodile vs Luvuvhu	159	20.00 (± 7.24)	2.76	*P* < 0.05	0.58 (± 0.11)	5.09	*P* < 0.0001
Crocodile vs Olifants	159	− 17.48 (± 7.98)	− 2.192	0.1705	− 0.17 (± 0.13)	− 1.39	*P* = 0.997
Crocodile vs Sabie	159	30.44 (± 7.98)	4.219	*P* < 0.001	0.67 (± 0.11)	5.98	*P* < 0.0001
Olifants vs Luvuvhu	159	− 37.48 (± 7.98)	− 4.699	*P* < 0.0001	− 0.76 (± 0.13)	− 6.02	*P* < 0.0001
Sabie vs Luvuvhu	159	10.14 (± 7.15)	1.42	*P* = 0.93	0.09 (± 0.11)	0.77	*P* = 1.00
Sabie vs Olifants	159	47.62 (± 7.87)	6.05	*P* < 0.001	0.84 (± 0.12)	6.85	*P* < 0.0001
**Time**							
Year	10	− 0.827 (± 0.89)	− 0.928	0.355	− 0.04 (± 0.01)	− 2.95	*P* < 0.01

**Fig. 3:**
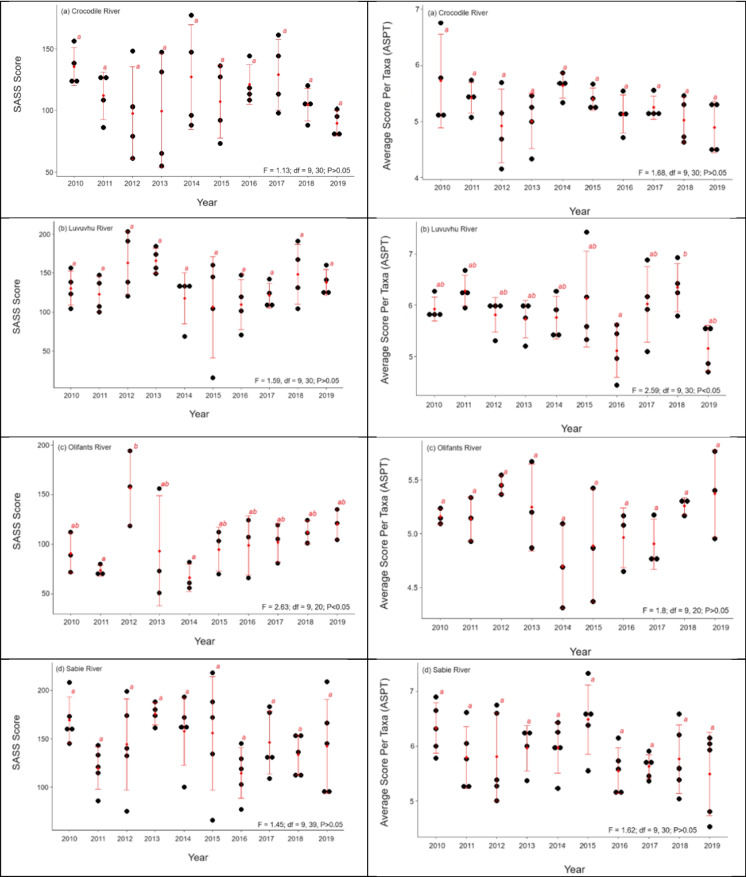
Mean (± std) for SASS scores and ASPT during the 2010–2019 river health study of four major rivers of the Kruger National Park including Crocodile, Luvuvhu, Olifants and Sabie rivers respectively, South Africa

Generally, the KNP rivers showed varying SASS scores over the study period, but this was not significant for Crocodile, Luvuvhu and Sabie rivers (Fig. 3), but there were some differences in the Olifants River particularly for the year 2012 which was different from 2011 and 2014 (Fig. 3). Although we observed no temporal differences in majority of the rivers for SASS scores, our results showed that there were some inter-annual differences that exist which were unique to each river. In the Crocodile River, year 2019, 2012 and 2013 showed the lowest mean SASS score and high during the year 2010, 2014 and 2017 (Fig. 3). Luvuvhu River SASS score was mostly stable with year 2014, 2015 and 2016 showing a slight decline when compared with the rest (Fig. 3). The Olifants River was the most disturbed or modified of all, were during the year 2010, 2011, 2013 and 2014, we observed the lowest SASS scores (Fig. 3). Sabie River recorded higher SASS scores during the study, but there were some notable years including 2011, 2016 and 2018 which showed a decline is SASS score (Fig. 3).

The Crocodile, Olifants and Sabie rivers showed no significant differences in ASPT over time (Fig. 3), but there were some differences in ASPT in Luvuvhu during the year 2016 and 2018 (Fig. 3). The different rivers attained their lowest and highest SASS scores in different years. For example, the lowest SASS scores were found in year 2011, 2013, 2014, 2016, 2015 and 2019 (Fig. 3) and whereas for ASPT it was year 2012, 2013, 2014, 2016 and 2019 (Fig. 3). Overall, the Olifants River was found to be the most modified river based on SASS score and ASPT, except in year 2012 and 2019 for both SASS score and ASPT (Fig. 3). Whereas Sabie River was found to be the least modified river of them all, with high SASS score and ASPT, except in year 2011, 2016 and 2018 for SASS Score and 2011, 2016 and 2019 for ASPT (Fig. 3).

The PES results showed that both the Sabie and Luvuvhu rivers were ranging between the ecological category A and C classes, based on their SASS scores, demonstrating that they were mostly above 60% intactness thus closely resembling their expected reference SASS scores (Table 2). Sabie River was relatively less modified only attaining class D in year 2011 and 2016, whereas the Luvuvhu River had class D in 4 years, i.e. 2010, 2011, 2014 and 2016 (Table 2). In contrast, the Crocodile and Olifants rivers ecological category was between class D to E, which was well below the 60% intactness mark, thus largely different and modified from their natural ecological state (Table 2). The Olifants River only had 1 year (i.e. 2012) where it was class C, of mostly class D and E (Table 2). In fact, it was the only river that attained class E throughout the study period, during the year 2011 and 2014 (Table 2). In contrast, Sabie River was the only river demonstrated class A and B, achieved in year 2013 and 2010, respectively (Table 2). In terms of the PES based on ASPT, only Sabie and Luvuvhu rivers had class A. Again, these were attained in different years; in Sabie River it was year 2010, 2013–2015 and Luvuvhu River in 2011, 2015, 2017 and 2018 (Table 2).

### Effect of environmental variables on SASS metrics

Multiple linear regression analyses showed that pH, EC, flow and habitat explained a significant 19% variation in SASS scores with pH, EC and flow showing a negative influence and whereas habitat was positive, but only EC was significant (Table 3). ASPT on the other hand showed to be negatively influenced by all measured environmental variables, with only EC shown to be significant. Collectively, all measured environmental variables explained 26.4% of the ASPT variability (Table 3).

**Table 3:**
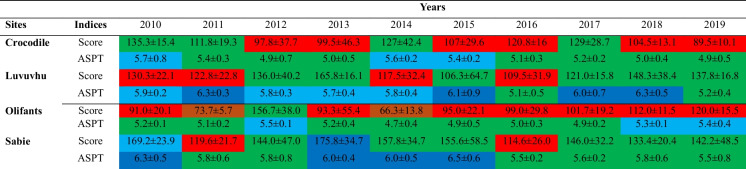
Mean (± standard deviation) of SASS5 score and ASPT across four KNP rivers (*n* = 4) over time (10 years). The dark blue colours denote Ecological category A (class A) where the SASS score and ASTP have retained 90 to 100% of its reference (or unmodified condition). The light blue colours denote Ecological category B (class B) where the SASS score and ASTP have retained 80 to 89% of the natural score. The green colours denote Ecological category C (class C) where the SASS score and ASTP have retained 60 to 79% of the natural score. The red colours denote Ecological category D (class D) where the SASS score and ASTP have retained 40 to 59% of the natural score. The brown colours denote Ecological category E or F (classes E and F) where the SASS score and ASTP have retained 0 to 39% of the natural score

## Discussion

Our long-term study used macroinvertebrate-based biomonitoring tool and selected physicochemical variables to assess the spatio-temporal variation of the river health and water quality for four KNP rivers. Our study showed a progressive deterioration of some rivers, namely Olifants and Crocodile rivers and departure from their expected health and water quality status. On the other hand, Sabie and Luvuvhu rivers showed an opposite trend, as they tended to have better river health status and water quality towards the end of our study. SASS scores and ASPT were found to be useful in determining the ecological health of KNP rivers both in time and space, with marked temporal variations which is attributed by catchment activities and events.

The KNP rivers exhibited a clear spatial variation in terms of their biotic indicators, with both Crocodile and Olifants rivers having the lowest SASS and ASPT scores, compared to their counterparts, Luvuvhu and Sabie rivers, with the highest scores. Simply put, Crocodile and Olifants rivers had the worst record of river health and water quality. Studying the macroinvertebrate diversity between the Sabie, Crocodile and Olifants river, Majdi et al. (2022) reported that Sabie River had the highest macroinvertebrates diversity while Crocodile River was the least (see also Riddell et al., 2019; Shikwambana et al., 2021). Using both upstream (outside the park) and downstream sites with the mainstem of Olifants river, Marr et al., (2017a, 2017b) showed similar observations with the current study, both in terms of water quality and biotic indicators. However, they further concluded that there were no material differences between upstream and downstream sites that were inside the park (Marr et al., 2017a, 2017b). Our results, together with those of Madji et al. (2022), contrast with this conclusion, as we observed an apparent increase in SASS and ASPT scores at downstream sites within the park, particularly from the western boundary toward the eastern regions. While we did not conduct a formal spatial trend analysis, this pattern suggests a potential beneficial effect of the park in improving river health (Zamora-Marín et al., 2016.

Given the formal protection the rivers receives from the park, i.e. preventing in-stream habitat and riparian alterations and given that these habitats remain or encouraged to be largely natural, species and functional diversity together with composition are expected to respond positively, and this can be reflective on freshwater systems especially if the water quality pressures also subdued (Riddell et al., 2019, 2022; Shikwambana et al., 2021). Although this sounds promising, there has been limited efforts when identifying and designing protected areas to be inclusive of freshwater systems (Abell et al., 2007; Nel et al., 2009). It has been proposed that freshwater focal areas, critical management zones and catchment management zones to be in cooperated into the existing protected areas framework (Abell et al., 2007). The current study will greatly benefit from some of these ideas in the cases of the Sabie and Luvuvhu rivers to sustain and maintain freshwater biodiversity and river health. In contrast, for systems like the Olifants and Crocodile rivers, active management interventions are required, such as the establishment of ecological buffer zones, implementation of freshwater protected areas (FPAs), and enhanced upstream catchment management to mitigate pollution and habitat degradation.

Similar observation were also noted with present ecological status (PES) results, as Crocodile and Olifants rivers had the highest levels of poor ecological condition results. The theoretical framework, development and implementation of PES in various countries are discussed by Noges et al. (2009) and Dallas (2013). Attainment of good ecological status is the objective of all water managers; however, this varies both spatially and temporarily (Lorenz & Field, 2013). In the present study, Luvuvhu and Sabie rivers were able to attain good ecological status at least for 4 years out of the 10, when considering the ASPT (Table 2). In fact, Sabie River had the highest ecological health of all the studied KNP rivers with the least years with poor category and majority best category. On the other hand, Crocodile and Olifants rivers were equally of the poorest ecological health as they had the highest record of poor category results. Attainment of good ecological status has been associated with good habitat quality, whereas urbanisation and nutrient pollution have been associated with poor ecological status (Lorenz & Field, 2013; Grizzetti et al., 2017).

The temporal effects were generally minimal, as with both SASS score and ASPT, only one river out of the four studied had a significant temporal variation. For example, no significant differences were observed in terms of SASS scores for all the rivers, except Olifants River, which had the lowest scores in year 2011 and 2014, and the highest scores in 2012. Similarly, using ASPT, only Luvuvhu River had a temporal variation between the years, with year 2016 and 2018 having the lowest and highest scores, respectively. In the year 2012, there was a major flooding which significantly affected the Olifants River (Mohlala et al., 2024). Further, in the year 2016 where ASPT was the lowest, the Luvuvhu River was heavily affected by the onset of El Nino Southern Oscillation (ENSO) phenomenon, which wrought extreme drought to the region and catchment (Malherbe et al., 2020; Mazibuko et al., 2021; Odiyo et al., 2015). The potential for biotic communities to respond and recover shortly after such events and in the case of persistent drought and flooding events in KNP Rivers (Weeks et al., 1996) and elsewhere (Pinha et al., 2016; Piniewski et al., 2017) still needs to be further investigated and understood.

Similar to the Luvuvhu River, the Olifants River had the lowest SASS score due to major flooding, but the year after 2012, the SASS score increased exponentially. These results and that of Luvuvhu River in 2017 and after demonstrate convincingly that macroinvertebrate can recover quickly, and this was also reflective on the SASS5 macroinvertebrates-based metrics. In the Sabie River, Weeks et al. (1996) demonstrated using both fish and macroinvertebrate communities that these taxa can recover rapidly from a drought. In the Olifants River, Mohlala et al. (2024) also revealed quick recovery and recolonisation after destructive flooding. Taken together, the apparent relatively quick recovery time of macroinvertebrates both in terms of upstream and downstream sites within the park, and temporary in terms of year-to-year scale is encouraging and reflective of the protected area’s potential to facilitate ecological recovery, restoration and protection of aquatic biodiversity (Acreman et al., 2020).

## Conclusion

This study clearly highlights the need for and importance of long-term monitoring studies and data as an evidence-based management strategy for freshwater resources to inform policy, particularly for iconic protected areas such as the KNP. It is also worth mentioning the river assimilation potential and macroinvertebrates recovery potential recorded in this study to be reflective in our results and thus worth highlighting. Thus, adding more evidence to the fact that protected areas or at least if freshwater resources are well maintained with ideal and wild habitat structure, quality and minimal anthropogenic inputs, these ecosystems will still have the ability to self-regulate and preserve aquatic integrity and biodiversity. This could be the case for the Sabie and Luvuvhu rivers. Unfortunately, the Olifants and Crocodile rivers were heavily modified by the upstream human and landscape developments, even though the park acted as a refugium to better the water quality and the SASS5 metrics downstream, these systems still came out to be poor in terms of the river health status as shown by the PES classifications. Considered the temporal aspect from this study, it clear that landscape activities will continue to be a challenge in these two catchments as opposed to the Sabie and Luvuvhu river catchments. Going forward, the present study recommends a large-scale ecological buffer zone before the rivers enters the park or implement freshwater protected areas (FPAs as proposed by Suski & Cooke, 2007). These FPAs are described as parts of the river partitioned in order to minimize disturbances and allow natural processes to take place. This conservation practice is well established in terrestrial and marine environments; however, its application to freshwater systems remains limited and relatively unexplored. As a result, freshwater conservation within protected areas remains poorly understood, presenting a valuable opportunity for future research to field-test and validate the effectiveness of such active interventions in improving river health and water quality. Furthermore, we recommend that these management practices be paired with continuous, annual biomonitoring to assess remediation outcomes. The Sabie and Luvuvhu rivers show promising recovery under current management, while the Olifants and Crocodile rivers may continue to degrade without targeted intervention. These projected trends can guide managers in prioritizing actions and allocating resources effectively. We also suggest further development of regional biomonitoring systems by enhancing macroinvertebrate-based indices like SASS5 through standardized protocols, regular training and data integration. Securing long-term funding and incorporating tools such as eDNA will support more scalable and robust monitoring.

## Supplementary Information

Below is the link to the electronic supplementary material.

Supplementary file1 (DOCX 651 KB)

## Data Availability

No datasets were generated or analysed during the current study.
