# Supplementary material for: Unravelling the long-term river health status of Kruger National Park Rivers using macroinvertebrate-based monitoring
Source: Environ Monit Assess. 2025 Jul 14;197(8):914. doi: 10.1007/s10661-025-14343-5 (PMC12259783; doi:10.1007/s10661-025-14343-5)
Supplement: Supplementary file 1 — Supplementary file1 (DOCX 651 KB) [file 10661_2025_14343_MOESM1_ESM.docx]

**Supplementary file1

Unravelling the long-term river health status of Kruger National Park Rivers using macroinvertebrate-based monitoring**

Hendrik Sithole^1*^, Samuel N. Motitsoe^2^, Thendo Mutshekwa^2,3,4^, Musa C. Mlambo^2,5^

^1^Conservation Services Division, Scientific Services, South African National Parks (SANParks), Kimberley, South Africa

^2^School of Animal, Plant and Environmental Sciences, University of the Witwatersrand, Private Bag 3, 2050, Johannesburg, South Africa

^3^Albany Museum, Department of Freshwater Invertebrates, Somerset Street, Makhanda (Grahamstown), 6139, South Africa

^4^Institute for Water Research, Rhodes University, Makhanda (Grahamstown) 6140, South Africa

^5^Rhodes University, Department of Zoology and Entomology, Makhanda (Grahamstown), 6140, South Africa

*Corresponding author

E-mail: [hendrik.sithole@sanparks.org](mailto:hendrik.sithole@sanparks.org)

| 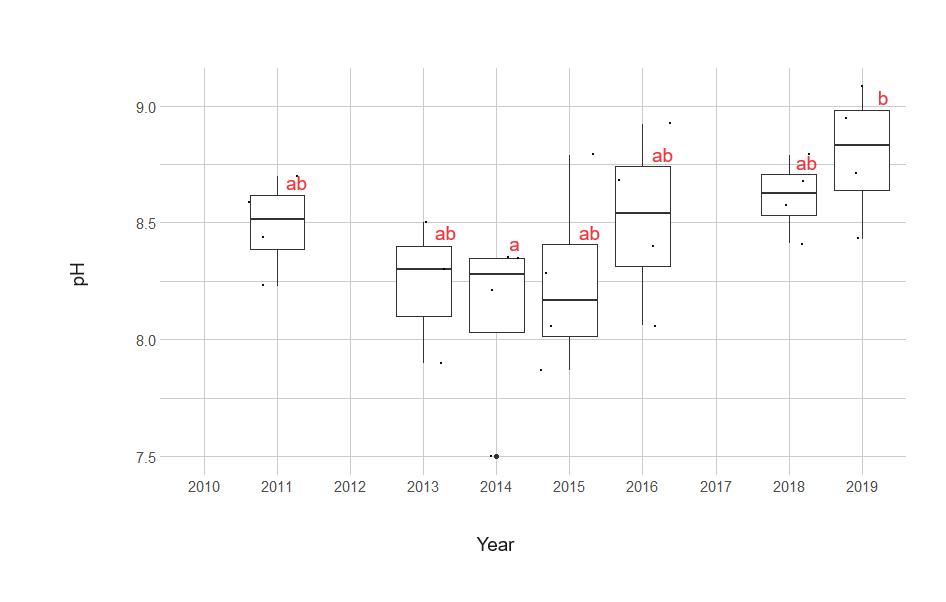 | 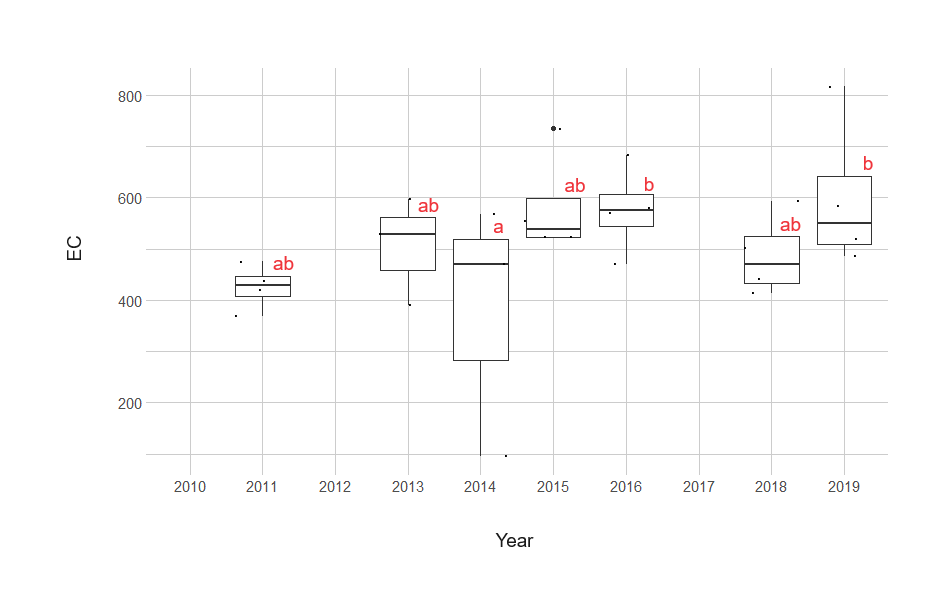 |
| --- | --- |
| 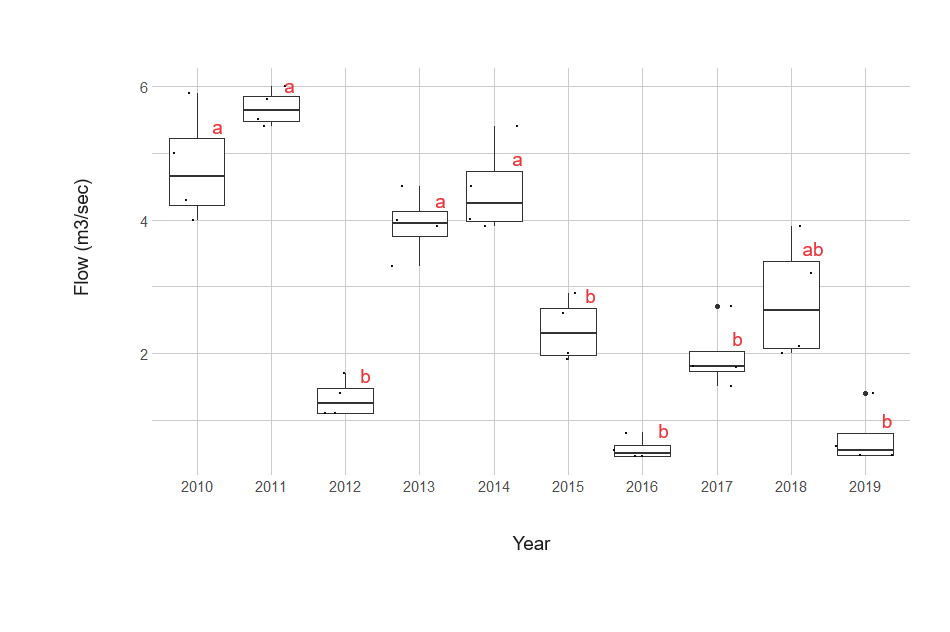 | 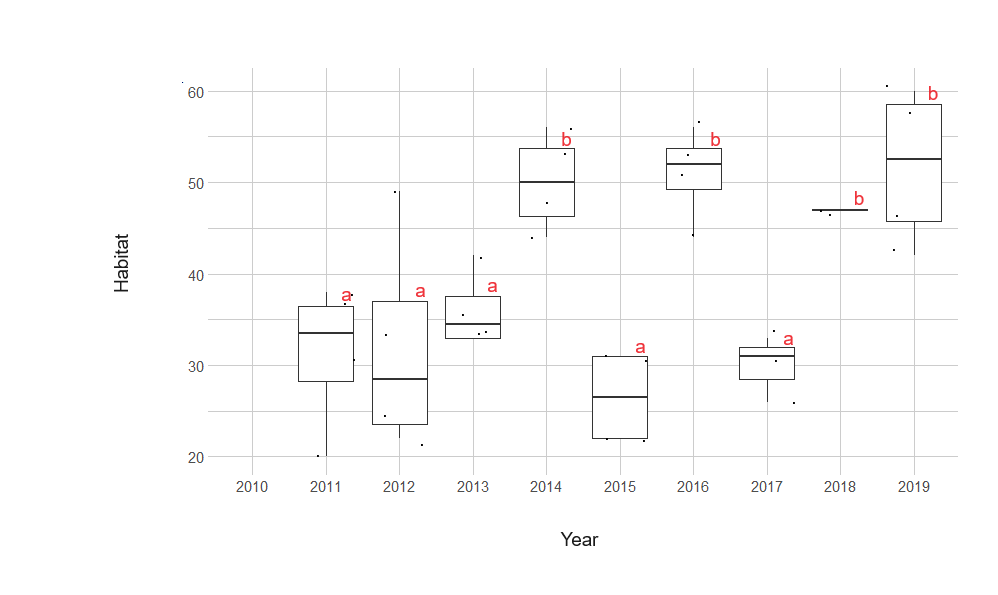 |

Figure S1: Crocodile rivers physicochemical parameters

| 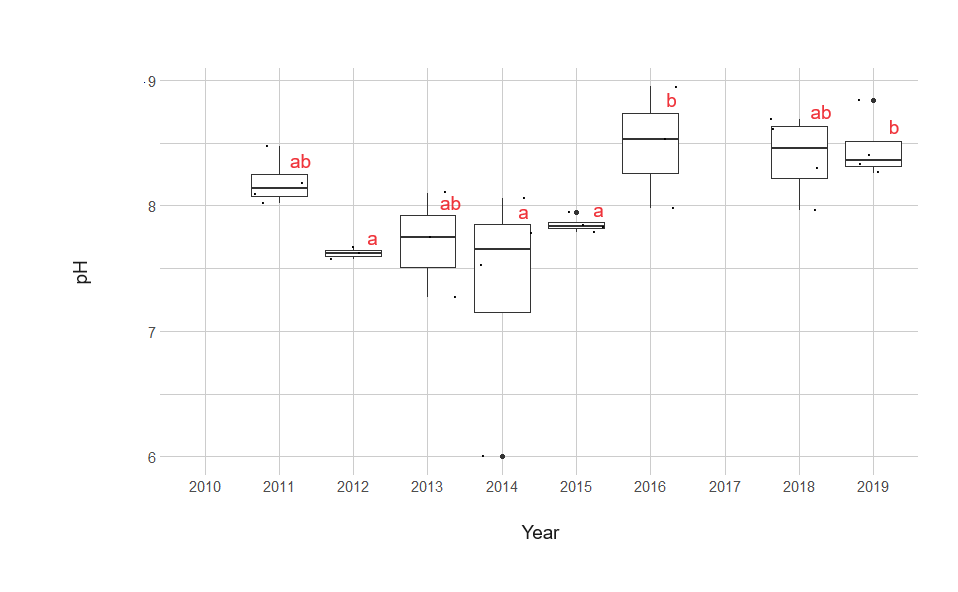 | 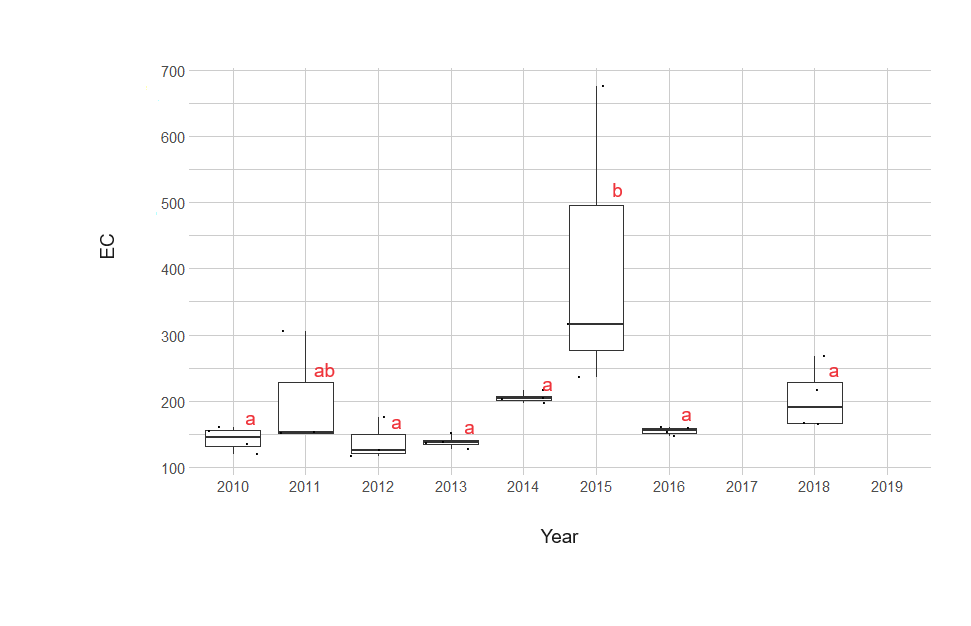 |
| --- | --- |
| 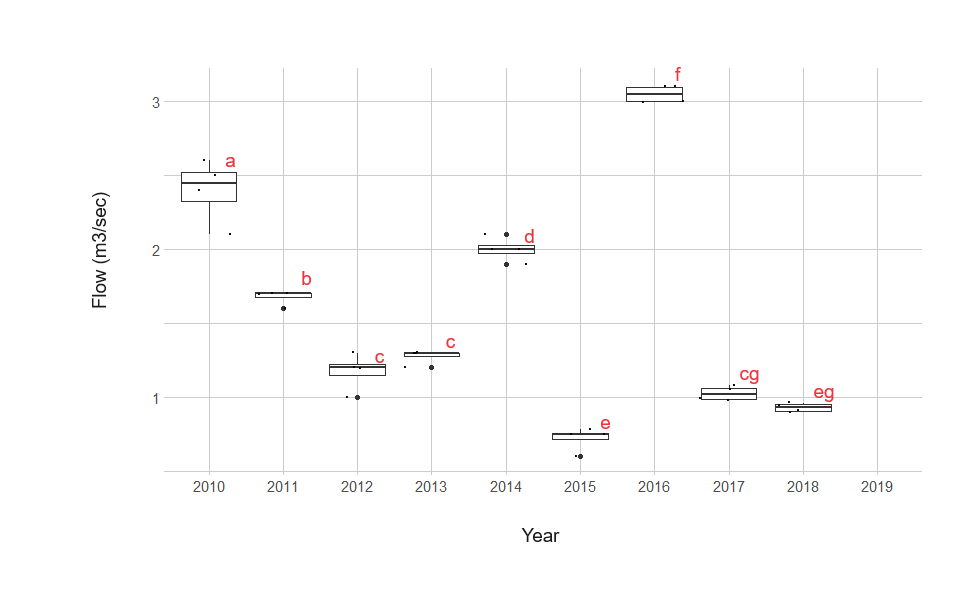 | 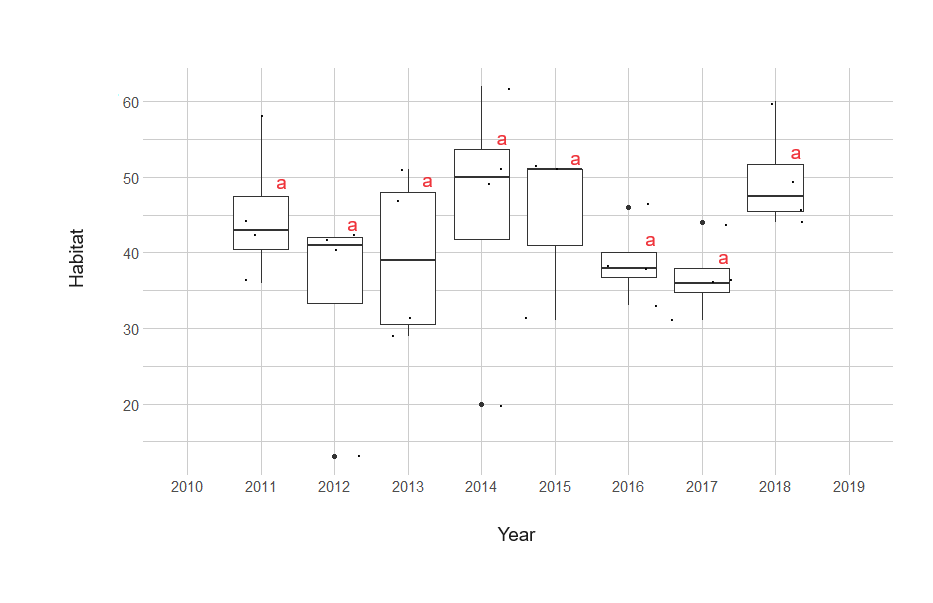 |

Figure S2: Luvuvhu rivers physicochemical parameters

| 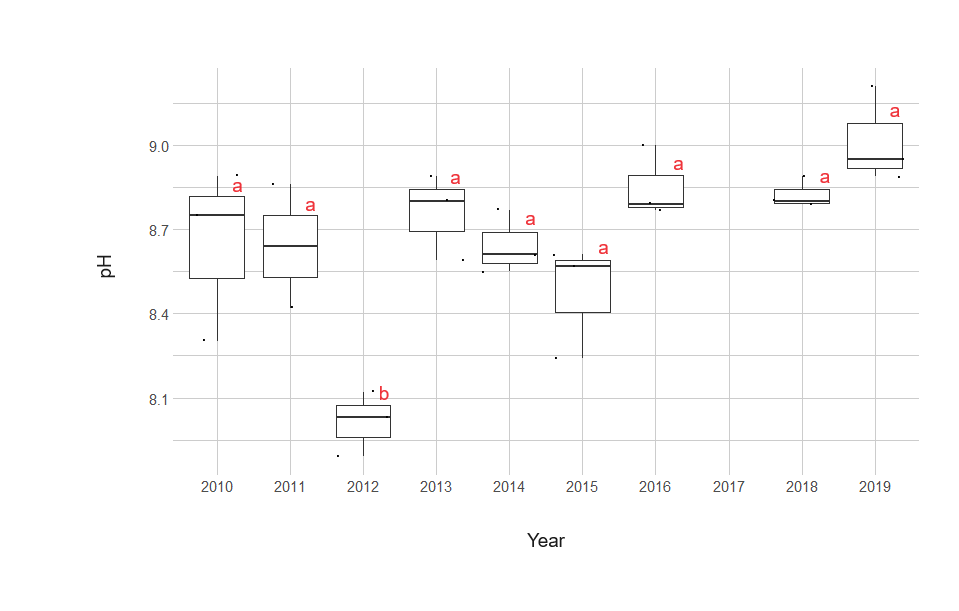 | 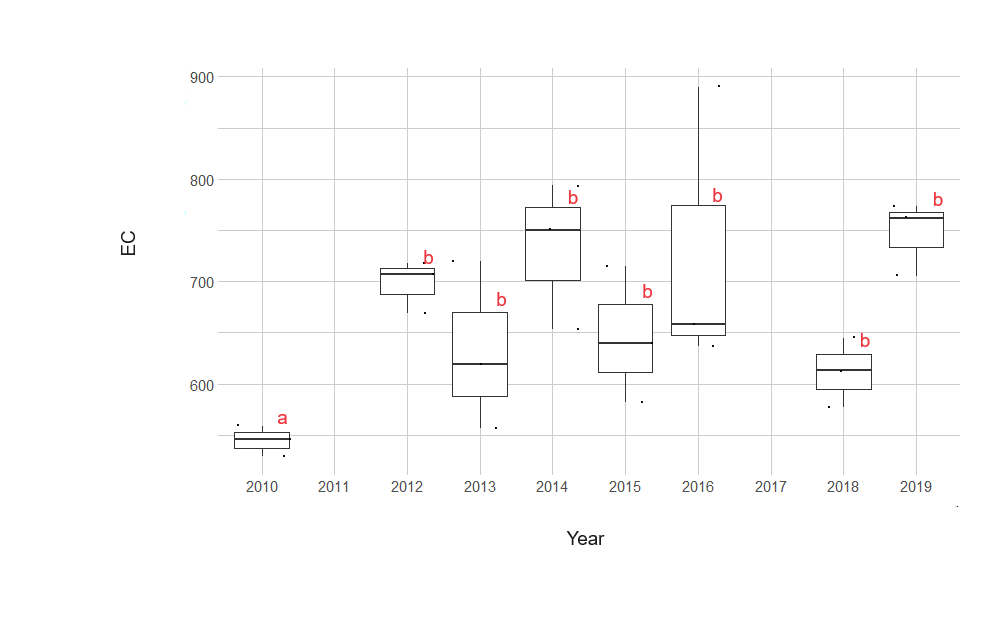 |
| --- | --- |
| 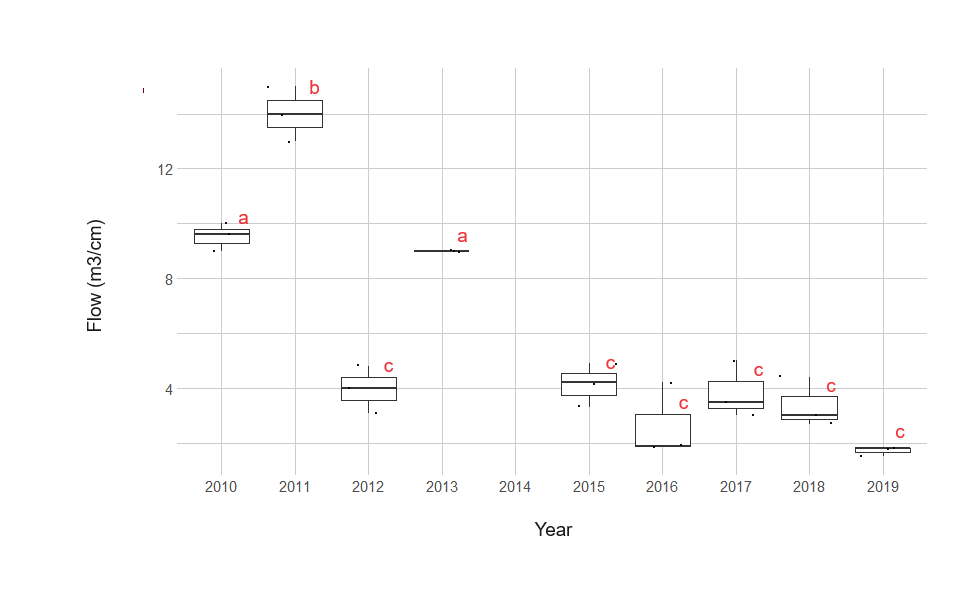 | 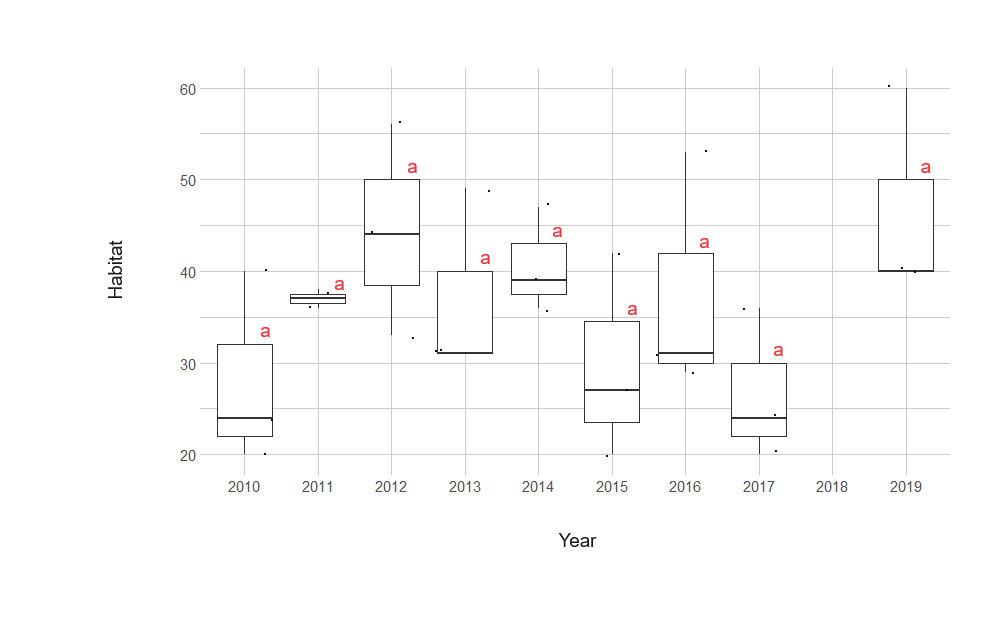 |

Figure S3: Olifants rivers physicochemical parameters

| 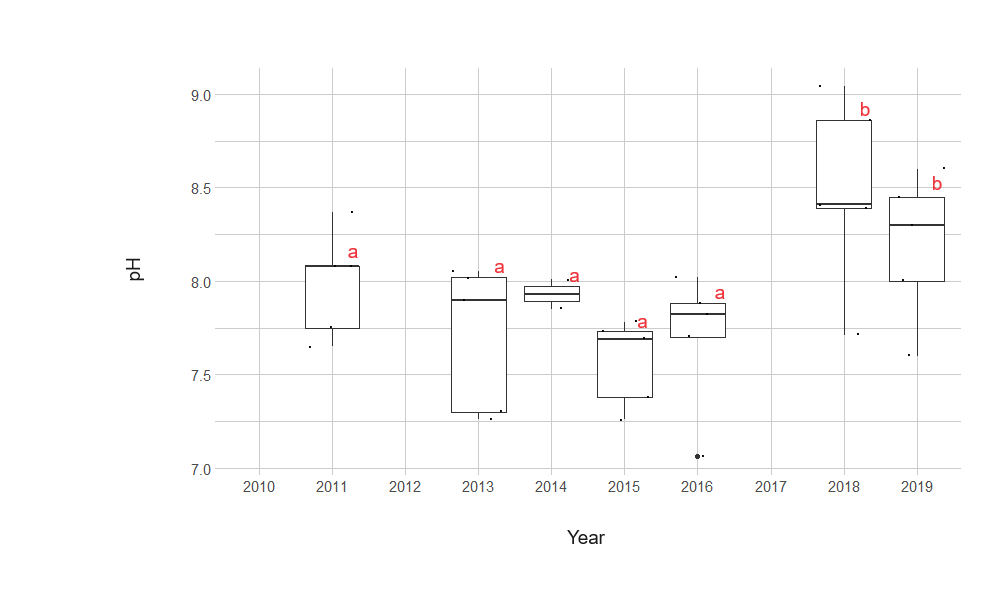 | 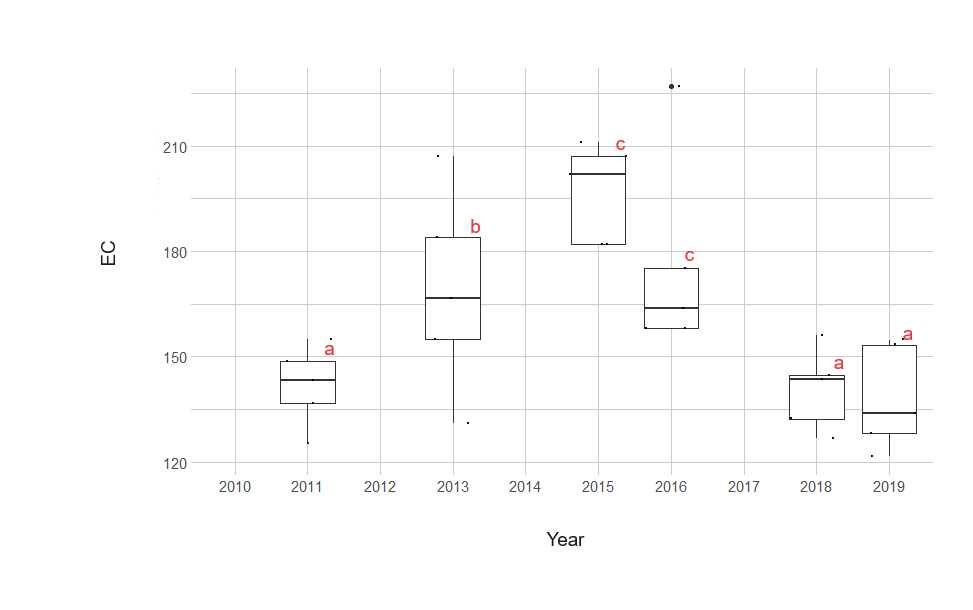 |
| --- | --- |
| 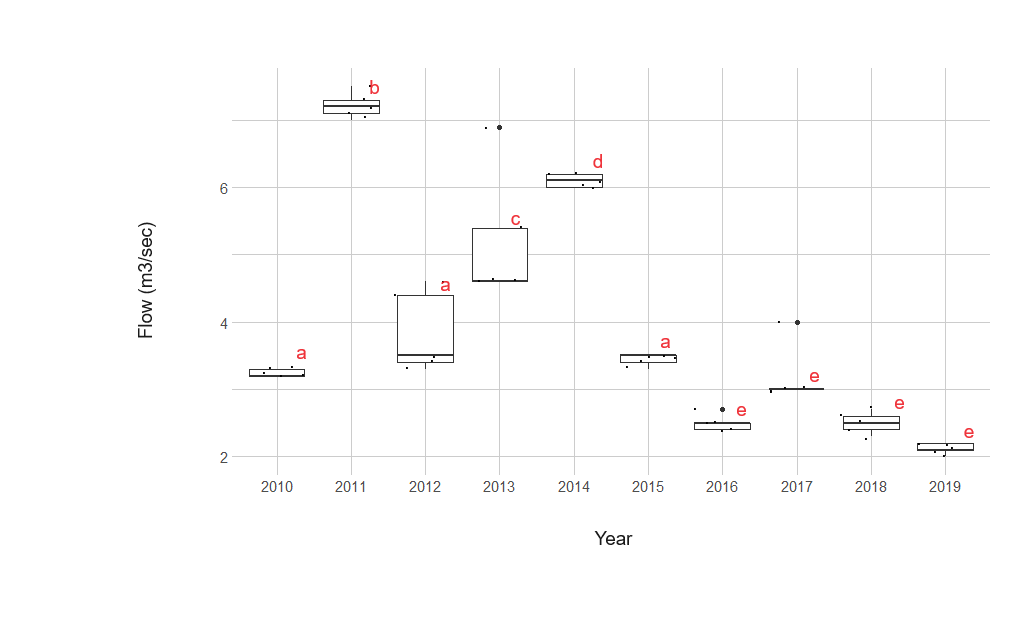 | 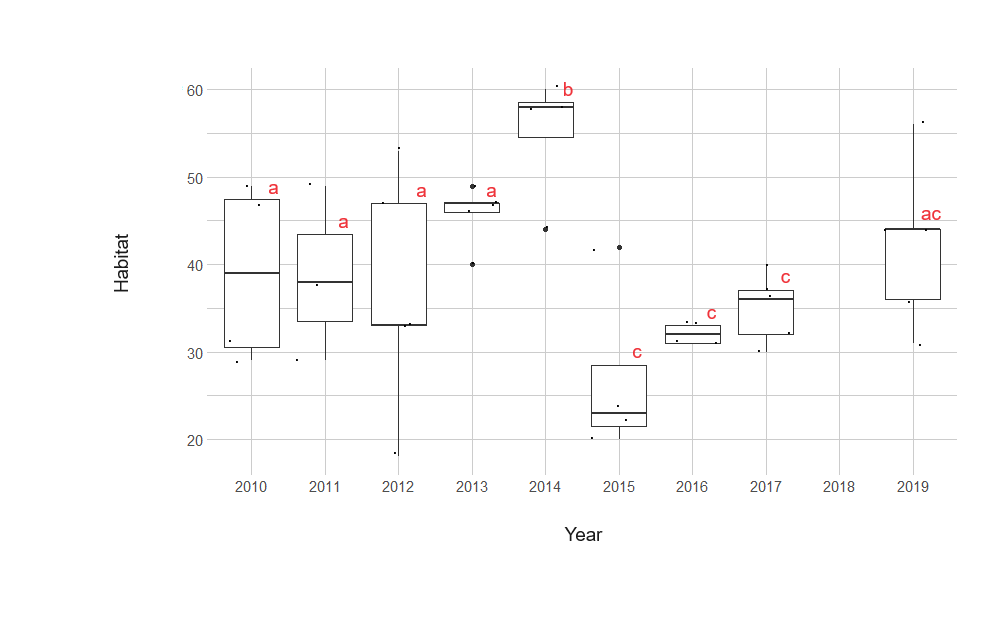 |

Figure S4: Sabie rivers physicochemical parameters
